# Supplementary material for: Development of a computer-aided design software for the quantitative evaluation of aesthetic damage
Source: PLoS One. 2019 Dec 18;14(12):e0226322. doi: 10.1371/journal.pone.0226322 (PMC6919621; doi:10.1371/journal.pone.0226322)
Supplement: S1 Code site — 1. (ZIP) [file pone.0226322.s001.zip › [EESC_jr][Projeto]Relatorio_07_21/vendors/select2/docs/_includes/examples/tags.html]

# Tagging support

Select2 can be used to quickly set up fields used for tagging.

Note that when tagging is enabled the user can select from pre-existing
options or create a new tag by picking the first choice, which is what
the user has typed into the search box so far.

orange
white
purple

{% highlight js linenos %}
$(".js-example-tags").select2({
tags: true
})
{% endhighlight %}
